# The effect of location and type of nutrition content claims (NCCs) on healthy food purchase intentions: findings from an experimental study

**DOI:** 10.1186/s12889-020-08761-y

**Published:** 2020-04-29

**Authors:** Shouwei Li, Ping Liu, Yan Guo

**Affiliations:** 1 Chengdu Agricultural College, No.392 Detongqiao Road, Wenjiang District, Chengdu, People’s Republic of China; 2 Business School, Sichuan University, No.24 South Section 1, Yihuan Road, Wuhou District, Chengdu, People’s Republic of China; 3 College of Information Engineering, Sichuan Agricultural University, No.46 Xinkang Road, Yucheng District, Yaan, Sichuan Province People’s Republic of China

**Keywords:** Nutrition content claim, Location effect, Purchase intention

## Abstract

**Background:**

Nutrition Content Claims (NCCs) are often used to enhance the appeal of healthy food products. Appropriate horizontal positioning of different NCCs in the consumer’s visual field may help to improve the effect of the claims. This study examines the extent to which NCCs on food packaging are effective depending on where the claims are located on the packaging and the type of claims.

**Methods:**

Guided by the location effect, a 2 (claim type: benefit-seeking vs. risk-avoidance) × 2 (claim location: left vs. right) experiment is conducted to investigate the influence of NCCs located on the left side of the observer’s visual field compared to claims on the right side of the observer’s visual field on purchase intentions when the claim is either benefit-seeking or risk-avoidance. The study was conducted online. A total of 400 participants took part in the experiment. The study obtained valid data from 365 participants (44.11% males). Analyses examined the purchase intentions of food products with different claims located in different locations. Differences were tested using a general linear model, and a level of significance of 0.05 was used.

**Results:**

The authors find that respondents show higher purchase intentions toward foods with risk-avoidance NCCs located on the left and toward foods with benefit-seeking NCCs located on the right side of the package.

**Conclusions:**

The results provide implications and suggestions for improving healthy food packaging and marketing strategies and for public health policy.

## Background

Many food advertisements make health- and nutrition-related (HNR) claims [1]. Although these claims share the objective of increasing the perception that a particular food has health benefits, they do so in very different ways. Food marketers have introduced three types of HNR claims in advertising: (1) health claims; (2) NCCs; and (3) structure/function claims [2–4]. Recently, there has been an influx of studies examining how food HNR claims can be used to influence consumers’ evaluations, intentions, and behaviors. HNR claims have been investigated at the micro-level, with single claims such as “low fat” [5–8]; at the macro-level, with broad descriptions such as “healthy” and “nutritious” [5, 9–11]; and at the meso-level, with classified descriptions such as “absence focus” [12]. However, while these studies have explored the impact of the different types of claims, few have considered the combined effect of claim types and claim display factors.

NCCs are the most frequently used HNR claim type in food advertising [1]. NCCs emphasize the specific enhancement of healthy nutrients or the reduction of unhealthy ingredients in food [2, 3]. These claims can be classified as benefit-seeking claims and risk-avoidance claims [2, 13] or presence-focus claims and absence-focus claims [12]. Benefit-seeking claims, or presence-focus claims, focus on positive attributes that are present in (or added to) the food while risk-avoidance claims, or absence-focus claims, focus on negative attributes that are absent (or removed) from the product. We focus on benefit-seeking and risk-avoidance NCCs.

In addition, in terms of location, some NCCs are placed on the left side of the observer’s visual field of the packaging while some are placed on the right side. Extant work relating to the location effect has shown that the location of the product picture affects consumers’ visual heaviness perception of the product [14], which results in the congruity of visual heaviness perception with product attributes, thus affecting consumers’ preferences and behaviors. For instance, positions on the right of the visual field of the package have more visual weight, meaning they attract the eye more and are “heavier” than the “lighter” positions on the left. For objects for which heaviness is regarded as a positive attribute, consumers have a preference for packages with the product picture located in heavy locations, while for objects for which heaviness is seen as a negative attribute, packages applying lighter locations for the product picture are preferred [14]. Similarly, a display with light (dark) colored objects located in an upper (lower) shelf location increases consumers’ perceptual fluency, resulting in the suggestion that “lighter” (“heavier”) positions are most suitable for light (dark) colored products [15].

This study focuses on the type and location of NCCs on food packaging. Considering that benefit-seeking and risk-avoidance claims create a difference in the weight perception of the emphasized ingredient and different visual heaviness perceptions result from different locations; thus, one important question arises: Would displaying a type of NCC on one side of the package lead to different purchase intentions than displaying it on the opposite side? Deng and Kanh [14] found that pictorial objects positioned on the right of the visual field carry more visual heaviness than when they are positioned on the left, hence the right in the visual field is the “heavy” location and the left is the “light” location. Consequently, the right (left) is a heavy (light) location. In this study, a food package is treated as the visual field and NCCs are treated as the pictorial objects.

Risk-avoidance claims emphasize the reduction of unhealthy ingredients [1]. Products with risk-avoidance nutrition claims tend to be estimated as lighter (in nutrient weight) by consumers and ingredients are perceived to be lighter when the claims are displayed in the left area of the visual field. Benefit-seeking claims focus on the enhancement of healthy nutrients [1]. Products with benefit-seeking nutrition claims tend to be estimated as heavier (in nutrient weight) and the ingredients are perceived to be heavier when the claims are displayed in the right area of the visual field. There is therefore consistent relevance between content claim type and location. In the packaging context, locating risk-avoidance claims on the left and benefit-seeking claims on the right would be congruent with consumers’ sensory correspondences, which are known as the tendency to associate a feature in one sensory modality with a feature in another, such as the association between sweetness and the sound of a piano or between bitterness and the sounds of brass instruments. We speculate that sensory correspondences exist between perceived nutrient weight by claim type and visual heaviness by claim location.

If claim type and location congruency conform to shoppers’ sensory correspondences, perceptual fluency should be enhanced. Further, a high level of perceptual fluency promotes consumers’ purchasing behaviors [16, 17]. Consequently, a display with risk-avoidance (benefit-seeking) NCCs on the left (right) would be more congruent with shoppers’ sensory correspondences than a display with risk-avoidance (benefit-seeking) claims on the right (left) and, as a result, would positively affect consumers’ food purchase intentions. Specifically, we propose the following hypotheses:

**H1a:** Benefit-seeking NCCs positioned on the right (left) of the visual field of the packaging result in higher (lower) food purchase intentions.

**H1b:** Risk-avoidance NCCs positioned on the left (right) of the visual field of the packaging result in higher (lower) purchase intentions.

We aim to investigate how to choose a more effective horizontal display location for different NCCs to enhance consumers’ purchase intentions toward healthy food, thus promoting healthy food consumption and public health. This work contributes both to the location effect by illustrating that NCCs’ horizontal locations interact with claim types to influence purchase intentions and to the NCC literature by introducing a way to alter shoppers’ behaviors. Our findings introduce a preferred display location for healthy food marketers to use for different types of NCCs. The following sections outline the resulting study. The paper concludes with a discussion of the study’s theoretical contributions as well as practical implications and future research directions.

## Methods

### Study design and participants

A two-factor (NCC location: right vs. left) × (NCC type: benefit-seeking vs. risk-avoidance) between-subjects experiment was employed. Milk was selected because benefit-seeking and risk-avoidance NCCs are both used on milk packages. According to the NCCs of milk in many marketplace scenarios, the benefit-seeking NCC in the experiment was determined as “high calcium” and the risk-avoidance NCC was manipulated as “low fat”. The fictitious brand name “Element” was selected for the study [18].

Four food product packages created by professional graphic designers were adopted. All four packages contained the product name, brand name, volume (ml), and a picture illustrating the product. Across different packages the claim type and location were varied. The “claim type and location” treatment was manipulated by displaying risk-avoidance NCCs on the left and benefit-seeking claims on the right visual field of the packing or benefit-seeking claims on the left and risk-avoidance claims on the right. Packages’ width/height ratio was 1.618, which is the “golden” ratio that has been widely applied in packaging.

Pretest 1 was applied to design the stimuli and test whether the stimuli had been manipulated successfully. Three key factors were considered: familiarity, visual complexity, and consumer attitudes toward the visual appearance of the product. Familiarity is the number of product-related experiences that have been accumulated by the consumer. The importance of familiarity in consumption is well established [19]. Visual complexity is the amount of detail or intricacy of the picture. The visual complexity of advertising plays a central role in influencing consumption behavior [20]. The visual appearance of food packaging will affect food attitude and purchase decisions [21]. Given these factors, participants were recruited through social platforms (*N* = 120, 42.50% male, *M*_age_ = 20.67, *SD* = 1.99), rated the stimuli, and confirmed no significant differences in terms of familiarity [*F* (3, 116) = 2.04, *P* = 0.112], visual complexity [*F* (3, 116) = 0.89, *P* = 0.450], and attitude [*F* (3, 116) = 0.74, *P* = 0.533] to the packaging. Two marketing experts examined the packages to ensure that they appear realistic and professional and are typical of packages shoppers actually receive, giving us confidence in the generalizability of the conclusions.

The sample size was calculated using G*power software version 3.1. The sample was calculated based on an effect size of 0.25, sample power of 0.95, and a significance level of 1%, resulting in 289 subjects. Thus, the final sample in the main study should include a minimum of 289 participants. The participants of the study were undergraduate students who had previously purchased milk.

### Data collection

A total of 400 undergraduates from a large Chinese university were recruited through social media platforms and received a nominal payment for taking part in the study. They were randomly assigned to one of four conditions in a two-factor (NCC location: right vs. left) × (NCC type: benefit-seeking vs. risk-avoidance) between-subjects design.

Participants were first given brief instructions and signed a consent form. They were then randomly assigned to one of the four experimental conditions and told to browse a package picture. After viewing the stimulus, they were asked to rate the familiarity, visual complexity, and attitude to the packages and were required to answer questions on their purchasing intentions toward the packaged food products. The participants’ age, gender, and milk consumption frequency were then collected and their health consciousness was measured. Health consciousness is an indicator of a consumer’s intrinsic motivation to maintain good health, and we measured this because it is related to how consumers react to health and nutrition information [22].

All variables were measured on a 7-point Likert scale ranging from 1 to 7 [23]. The scores of multiple items were averaged to form a single measure. Familiarity with the packaging was measured through three items: unfamiliar/inexperienced/not knowledgeable (1) to familiar/experienced/knowledgeable (7) where *α* = 0.893 [24]. Package visual complexity was measured by one item: “very simple (1) to very complex (7)” [20]. Attitude toward the package was measured through three items: bad/dislike/not nice (1) to good/like/nice (7) where *α* = 0.854 [25]. Purchase intent was measured by three items: unlikely/improbable/impossible (1) to likely/probable/possible (7) where *α* = 0.913 [25]. Health consciousness was measured by the statements “I try to avoid foods that are high in fat,” or “I am concerned about getting enough calcium in my diet,” with a response range of definitely disagree (1) to definitely agree (7) [22].

Ethical approval was exempted by the medical ethics committee of Sichuan University Institutional Review Board (http://yxglc.scu.edu.cn/info/1012/1116.htm). Informed written consent to participate in the study was obtained from all participants.

### Data analysis

Mean (M) ± Standard Deviation (SD) and 95% Confidence Intervals (CI) were used to describe the continuous variables. A one-way Analysis of Variance (ANOVA) was used to compare package familiarity, package visual complexity, package attitude, and health consciousness among different subgroups. A two-way ANOVA was used to compare purchase intentions among different subgroups. Statistical Package for the Social Sciences software (SPSS, Version 22.0) was used.

## Results

### Characteristics of the participants

The response rate was 100%. All 400 participants’ responded that they had purchased milk in the past; however, the final sample consists of 365 participants after 16 subjects dropped out of the study and 19 subjects were excluded for issues such as poor response time or incorrectly answered validation questions. No significant differences were found in age, sex, or milk consumption frequency between participants in the four experimental conditions, as shown in Table 1.

**Table 1:** Participant characteristics

	Total sample (*n* = 365)	Benefit-seeking		Risk-avoidance	
		Left (n = 91)	Right (*n* = 93)	Left (*n* = 90)	Right (*n* = 91)
	*M* (*SD*) or %	*M* (*SD*) or %	*M* (*SD*) or %	*M* (*SD*) or %	*M* (*SD*) or %
Age	20.83 (2.03)	21.25 (2.10)	20.90 (2.09)	20.37 (1.97)	20.82 (1.87)
Sex (male)	44.11	42.86	46.24	43.33	43.96
Milk consumption frequency					
Never	0	0	0	0	0
Seldom	11.78	10.99	9.68	10.00	16.48
Sometimes	33.15	31.87	35.48	33.33	31.87
Often	39.73	39.56	41.94	43.33	34.07
Very often	15.34	17.58	12.90	13.33	17.58

### Package familiarity, visual complexity, attitude and health consciousness

The results, provided in Table 2, show that no significant differences exist among package familiarity [*F* (3, 361) = 1.70, *P* = 0.168], visual complexity [*F* (3, 361) = 0.73, *P* = 0.534], attitude [*F* (3, 361) = 0.39, *P* = 0.761], and health consciousness [*F* (3, 361) = 1.21, *P* = 0.306].

**Table 2:** Mean difference of variables by experimental condition in Study

	*N*	Package familiarity		Package visual complexity		Package attitude		Health consciousness		Purchase intention	
		*M*	*SD*	*M*	*SD*	*M*	*SD*	*M*	*SD*	*M*	*SD*
Benefit-seeking											
Left	91	4.84	1.22	3.44	1.20	4.43	1.15	4.99	1.16	4.33	1.11
Right	93	4.85	1.25	3.61	1.25	4.26	1.23	4.80	1.36	4.9	1.15
Risk-avoidance											
Left	90	4.77	1.17	3.46	1.15	4.40	1.24	4.67	1.23	4.58	1.23
Right	91	4.49	1.21	3.65	1.16	4.31	1.26	4.70	1.24	3.84	1.10

### Purchase intention

An analysis of purchase intentions toward the packaged food as a function of nutrition claim location and type yielded significant two-way interaction [*F* (1, 361) = 29.47, *P* = 0.000]. As hypothesized in H1a and H1b, participants in the experimental condition of benefit-seeking claim reported a greater purchase intent when the NCC was located right in the visual field rather than left in the visual field [*F* (1, 362) = 10.90, *P* = 0.001]; however, for participants in the experimental condition of risk-avoidance NCC, scoring for purchase intentions of a product with left-located claims is significantly higher than that of right-located ones [*F* (1, 362) = 18.50, *P* = 0.000] (Table 2). Thus, hypotheses H1a and H1b are supported.

## Discussion

This study is one of the first experimental studies, to our knowledge, that has assessed the impact of types of NCCs and their horizontal locations on the package on consumers’ healthy food purchase intentions. The study results demonstrate that benefit-seeking (risk-avoidance) NCCs positioned on the right (left) of the visual field of the packaging result in higher food purchase intentions. While NCCs can provide important information for consumers, they do not always promote healthy food consumption. Based on the location effect theory, this study identifies a way to promote the consumption of healthy food by enhancing the effect of NCCs. Our findings enrich the growing body of literature on the application of food HNR claims to affect consumer behavior.

Existing research mainly discusses how to use benefit-seeking or risk-avoidance claims from the perspective of consumer motivation [2] and regulatory focus [26]. However, two important points have been neglected. First, the NCC type is sometimes determined by food characteristics. For example, “high calcium” is often advertised on milk packaging rather than “low calcium,” and biscuits packaging often advertises “low sodium” instead of “high sodium.” Therefore, when the claim type is determined by the food itself, it is important to determine how to display the claims. Second, the difference of the weight perception of the ingredients in benefit-seeking or risk-avoidance claims is easy to ignore but these small differences may also impact consumers’ perceptions. This study supplements and contributes to the literature in the above two aspects.

More broadly, this work adds to the increasing literature on the location effect [14, 15, 27]. Prior research has demonstrated the location effect in the domains of product image [15, 28], brand logo [29], and food product display [30]. Moreover, some have shown that nutrients located nearer to the label’s top receive more visual attention from consumers [31]**.** Our research is the first to establish the horizontal location’s effect of different types of NCCs on food purchase intentions. We find that, for food products, there is no pervasive preference for “heavy” or “light” positions but that the location of the NCC should depend on the type of claim. This is consistent with the finding that a “healthy-left, unhealthy-right” presentation pattern results in a relatively higher probability of choosing healthy options compared to a “healthy-right, unhealthy-left” display pattern [30].

Our research has interesting implications for package designs and public health. For instance, if a food marketer wants to increase sales of a product with risk-avoidance (benefit-seeking) NCCs, the marketer should make the claim on the left (right) of the visual field of the package. In addition to NCC design elements, to minimize possible cognitive resource depletion, food marketers should adapt the design elements to make consumers’ purchase behaviors less perceived confusion and more efficient. For example, marketing cues placed where they are congruent with the cue type are expected to enhance product sales or average spending per customer. Finally, public health policymakers and health organizations should focus on design elements as it is a relatively easy way to promote the consumption of healthy products. Simultaneously, they should encourage healthy food marketers to match the design of the NCC type and claim location on food packaging to promote the choice of healthier food products, thus benefiting public health.

As with all research, this study has limitations. First, the researchers selected participants using a convenience sample from a university in China. Further research should be conducted to expand the findings of the current research to different populations to increase generalizability. Second, the study in this paper is an explorative experiment. The findings are limited to particular food products and specific NCCs and do not reflect a full array of food categories. Future research could expand products and NCCs. Furthermore, this study has focused on the influences of left and right locations on NCCs. Future research could explore the implications of other claim locations or formats, for example vertical (top-bottom) displays of benefit-seeking and risk-avoidance claims. Moreover, NCCs on food packages may bias food healthiness [32, 33] and tastiness perceptions. Therefore, determining whether different types of NCCs in different locations affect consumers’ healthiness and tastiness perceptions would have relevant managerial significance and be conceptually interesting.

## Conclusions

In this research, we explored the effects of two design elements on food packaging—the location of the NCC and the type of claim—on consumers’ purchase intentions. The study provides evidence that these two design elements can be applied strategically to increase healthy food purchase intentions. Specifically, we confirmed that for food products with benefit-seeking NCCs, a nutrition claim placed on the right is preferred whereas for products with risk-avoidance claims, a nutrition claim on the left is preferred.

## Data Availability

The data analysed during the current study are available from the corresponding author on reasonable request.

## Abbreviations

NCC: Nutrition Content Claim
HNR: Health- and nutrition-related
SD: Standard Deviation
M: Mean
CI: Confidence Intervals
ANOVA: Analysis of Variance
